# White Matter Brain Network Research in Alzheimer’s Disease Using Persistent Features

**DOI:** 10.3390/molecules25112472

**Published:** 2020-05-27

**Authors:** Liqun Kuang, Yan Gao, Zhongyu Chen, Jiacheng Xing, Fengguang Xiong, Xie Han

**Affiliations:** 1 School of Data Science and Technology, North University of China, Taiyuan 030051, China; 18335162630@163.com (Y.G.); 15079561913@139.com (Z.C.); hopenxfg@nuc.edu.cn (F.X.); 2 School of Software, Nanchang University, Nanchang 330047, China; xingjiacheng628@163.com

**Keywords:** Alzheimer’s disease (AD), persistent homology, graph theory, brain network, diffusion tensor imaging

## Abstract

Despite the severe social burden caused by Alzheimer’s disease (AD), no drug than can change the disease progression has been identified yet. The structural brain network research provides an opportunity to understand physiological deterioration caused by AD and its precursor, mild cognitive impairment (MCI). Recently, persistent homology has been used to study brain network dynamics and characterize the global network organization. However, it is unclear how these parameters reflect changes in structural brain networks of patients with AD or MCI. In this study, our previously proposed persistent features and various traditional graph-theoretical measures are used to quantify the topological property of white matter (WM) network in 150 subjects with diffusion tensor imaging (DTI). We found significant differences in these measures among AD, MCI, and normal controls (NC) under different brain parcellation schemes. The decreased network integration and increased network segregation are presented in AD and MCI. Moreover, the persistent homology-based measures demonstrated stronger statistical capability and robustness than traditional graph-theoretic measures, suggesting that they represent a more sensitive approach to detect altered brain structures and to better understand AD symptomology at the network level. These findings contribute to an increased understanding of structural connectome in AD and provide a novel approach to potentially track the progression of AD.

## 1. Introduction

Alzheimer’s disease (AD) [1] is a common neurodegenerative disease in the elderly. Clinical manifestations are mainly memory dysfunction and cognitive decline. As a transitional stage between normal aging and AD, mild cognitive impairment (MCI) increases the risk of developing dementia. Understanding the physiological deterioration caused by AD and MCI provides an opportunity to develop future drugs and predict AD onset [2,3]. The human brain is interconnected by a large number of neurons through synapses, forming a highly complex network system that realizes various intelligent behaviors of human beings. Within these neural networks, even minor mutations can cause serious diseases [4]. In the past several years, some literatures [5,6,7] have begun to explore the treatment of AD and develop drugs from the perspective of structural brain network reconstruction and neuronal circuit repair [8]. Many postmortem histological and in-vivo imaging studies indicate widespread white matter (WM) changes in patients with MCI and AD [9,10]. WM brain network research provides a chance to understand how abnormal structural connections can lead to cognitive and behavioral deficits in these patients.

Graph theory [11] has become an important tool for studying the progression of AD which is characterized by network disruptions that seem to reflect the spread of pathological changes in the brain [12]. Some neurobiologically meaningful topological features of graph theory have been used to measure and evaluate the integration and segregation of the brain network, including small-world attributes, hubs, modularity, etc. [13]. By comparing the abnormal changes in these topological properties, the pathophysiological mechanisms of AD have been revealed to a certain extent, such as a loss of small-worldness and a redistribution of hubs [14,15]. Although graph theory can find quantitative biomarkers of brain disease, it is necessary to threshold the brain network in advance [16], which will alter inferred network topologies and thus produces inconsistent results based on different thresholds [17]. Moreover, there are no widely accepted criteria for choosing thresholds.

As a mathematical tool in algebraic topology, persistent homology [18] has been developed for the analysis of topological data, including brain networks. It establishes a multiscale brain network over all possible thresholds by graph filtration [19], and the brain networks at each scale are nested with each other, which not only provides an important framework for the analysis of the evolution of the characteristics of the brain network, but also identifies the persistent and robust structures from noise during network dynamics. Unlike the brain network studies on spatiotemporal dynamics [20,21], it only considers the intrinsic dynamics of network nodes without time delays. Using a typical persistent feature, Betti number plot (BNP) [22], some have found abnormal functional brain networks in the study of epilepsy [23], autism spectrum disorder, and attention-deficit hyperactivity disorder [22,24] and others have reported disrupted structural brain networks in maltreated children [25]. In our prior study [26], we proposed an integrated persistent feature (IPF) based on BNP and found that it has robust statistical capability in the study of functional brain network of AD and MCI in multiple brain atlases of different sizes. Although some persistent homology-based measures have been proposed to characterize the global organization of brain network, they have never been applied to measure the AD-related structural brain networks so far.

In this study, we investigate the integration and segregation of the WM brain connectome. We hypothesize that the alterations of AD progression could be understood through measuring the global structural network, and the persistent homology-based methods may provide additional insights into AD symptomology beyond studies of graph theory. We hope this study could provide a novel approach to potentially track efficacy of drug treatment for Alzheimer’s disease in terms of medical practice. With the cross-sectional diffusion tensor imaging (DTI) data of 40 AD, 77 MCI and 33 normal controls (NC), we set out to test this hypothesis by computing some standard graph-theoretic measures and our suggested persistent features for each subject’s WM network and then comparing their statistical differences across the three groups to reveal AD symptomology at the network level to some extent.

## 2. Results

### 2.1. Demographic Information

There were 150 individuals aged from 56 to 90 participated in this study from the Alzheimer’s Disease Neuroimaging Initiative (ADNI) database (adni.loni.usc.edu) [27]. Since the number of NC was less than the number of patients in DTI dataset, we used all 33 NC subjects and matched them to 77 MCI and 40 AD patients by gender, age, and education (Table 1).

As shown in Table 1, there were no significant differences in gender, age, and education across three groups using nonparametric Kruskal–Wallis test [28], while there were significant difference in clinical dementia rating (CDR) global scores [29]. Diagnostic classification was made by ADNI investigators using established criteria [30]. In addition, the displacement at any direction of the head movement in all the studied images was less than 1 mm, and the rotation at any angle was less than 1°.

### 2.2. WM Brain Network

**Figure 1:**
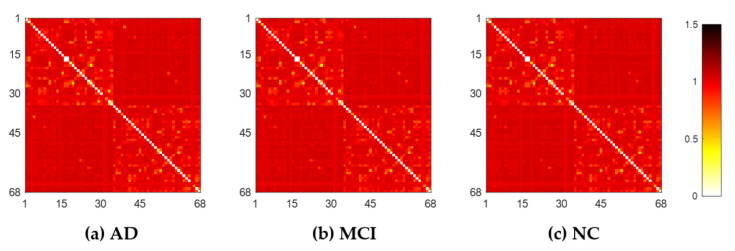
The original weighted white matter (WM) networks of the three groups, AD (**a**), MCI (**b**), and NC (**c**), where color bar shows the weights between any pair of region-of-interests (ROIs).

**Figure 2:**
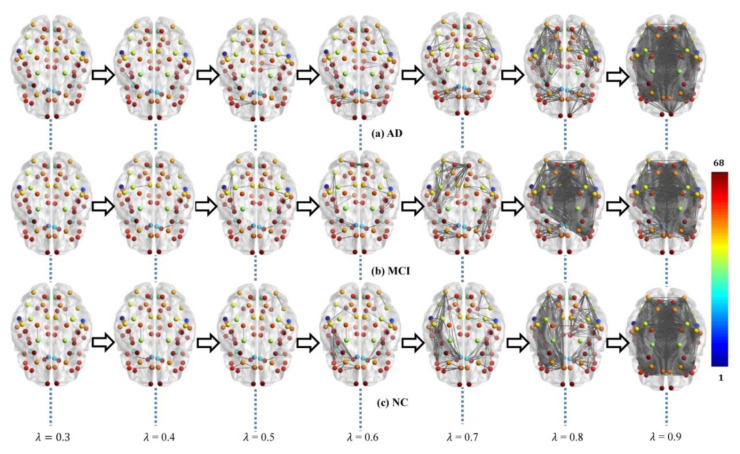
Multiscale WM networks of the three groups, AD (**a**), MCI (**b**), and NC (**c**), at some filtrations where color bar shows the ROI index. The AD group shows more sparse connections (i.e., more segregated connected components) comparing to controls when the filtration values are smaller (*λ* ≤ 0.8).

Each subject’s original weighted network was constructed based on Desikan–Killiany brain atlas [31] with 68 (DK68) region-of-interests (ROIs), using 1 minus Pearson correlation as described in Section 4.2. The adjacent matrices of three groups of mean weighted networks are shown in Figure 1. All edge weights were included and were ranged from 0 to 1.3 in our experiment, although theoretically they can be between 0 and 2. The lower weight represents the stronger association between ROIs. From Figure 1, there were no significant visual differences between the three groups of original networks. In order to seek their differences, we further constructed multiscale WM networks (Figure 2) from original networks using graph filtration [19] at all possible scales for studying persistent features. We found AD had more segregated connected components (sparser connections) at most filtration points before aggregating to the giant component, comparing to NC.

### 2.3. Network Properties

**Figure 3:**
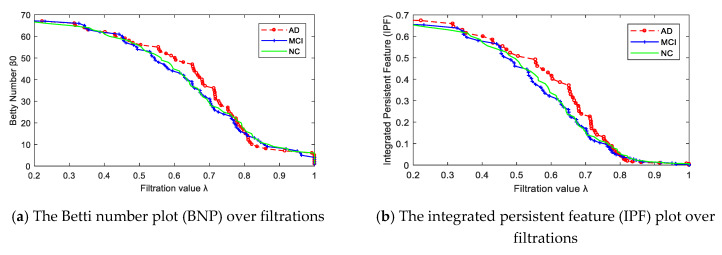
The persistent features plots of Betti number *β_0_* (**a**) and IPF (**b**) for three groups of mean networks.

After constructing multiscale WM networks, we plotted two persistent features: Betti number *β*_0_ and IPF over all filtration values. Figure 3 shows the dynamic plots for the mean networks of AD, MCI, and NC groups. Consistent with Figure 2, there were more separately connected components in AD than MCI and NC at most filtration values, since the AD curve was above the MCI and NC curves most of the time, indicating a more segregated organization structure in AD.

**Figure 4:**
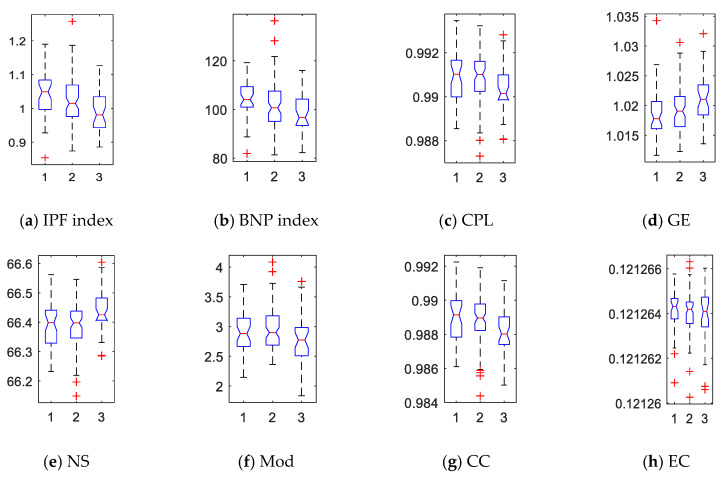
Box plots of network indices for 150 subjects across the three groups. Here, 1, 2, and 3 represent AD, MCI, and NC, respectively. Network indices: (**a**) IPF—integrated persistent feature; (**b**) BNP—Betti number plot; (**c**) CPL—characteristic path length; (**d**) GE—global efficiency; (**e**) NS—nodal strength; (**f**) Mod—modularity; (**g**) CC—clustering coefficient; and (**h**) EC—eigenvector centrality.

The absolute slope of each curve was refined into a network index. We computed all subjects’ IPF and BNP indices and show their box plots in Figure 4a,b where 1, 2, and 3 represent AD, MCI, and NC, respectively. In general, the IPF and BNP indices in AD group were higher than MCI, and much higher than NC. In addition, we computed the traditional network properties based on graph theory to investigate the integration, segregation, centrality, and other characteristics in the brain connectome. Specifically, the characteristic path length (CPL), global efficiency (GE), nodal strength (NS), modularity (Mod), clustering coefficient (CC), and eigenvector centrality (EC) of each subject’s WM network were quantified, as shown in Figure 4c–h. We saw CPL, Mod, and CC were higher in the patient groups, while GE and NS were lower. However, the difference of EC among three groups was hard to observe.

### 2.4. Statistical Group Difference Performance

Using different network indices, the statistical difference of all 150 individual networks across AD, MCI, and NC were obtained in Table 2. Our IPF got most significant differences in AD vs. MCI vs. NC (*p* = 0.002), AD vs. NC (*p* = 0.0003), and MCI vs. NC (*p* = 0.007). It almost got a significant difference between AD and MCI (*p* = 0.084). Another persistent index BNP also obtained great performance in statistical inference, only slightly weaker than IPF. Furthermore, all network indices detected significant differences between AD/MCI and NC (*p* < 0.05), except for EC which didn’t obtain significant difference between MCI and NC. The difference between AD and MCI couldn’t be detected by any graph theoretic measures. In general, our IPF shows better statistical power than another persistent feature BNP, and is much better than traditional graph-based features, CPL, GE, NS, Mod, CC, and EC.

### 2.5. Main Findings

Our study is the first to assess how persistent homology-based features characterize the abnormal global organization in structural network of AD. We have three main findings in investigating the integration and segregation of AD-related brain networks at the global network level.

First, we found abnormal integration of global network organization was presented in MCI, especially in AD, comparing to controls. The patient groups showed significantly increased CPL and reduced GE and NS using graph theory. In the brain dynamics by graph filtration based on persistent homology, the BNP and IPF indices of AD were significant different from MCI and NC (AD > MCI > NC), suggesting the altered aggregation pattern in patient groups. This finding is consistent with most of existing studies [32,33] as AD and MCI have less integration typically in whole brain organization.

Second, we found more highly segregated organization in MCI and AD comparing with NC, providing an evidence of AD as a disconnection syndrome [12]. The patients demonstrated more separated components (larger Betti number $\;\beta_{0}$) in global network. This study introduces a novel perspective of persistent homology that confirms the increased segregation in AD structural networks.

At last, it is first time to study structural brain network of AD using persistent homology-based methods which achieved stronger power in statistical inference among three groups (*p* < 0.01) than standard graph-theoretic measures. In our previous AD-related brain network studies on fluorodeoxyglucose positron emission tomography (FDG-PET) [34] and resting-state functional magnetic resonance imaging (MRI) [26], we also exhibited the superiority of persistent homology comparing to graph theory. Taken together, persistent homology may be a more sensitive mean to detect altered brain structure and understand AD symptomology at the network level.

## 3. Discussion

### 3.1. Validation on Various Parcellations

In general, the network measures are sensitive to the brain parcellation strategies [2,35]. In order to evaluate the robustness of our findings, we performed identity experiments on other three parcellation schemes. Specifically, a subcortical parcellation was performed from a manually labeled training dataset [36], including eight subcortical structures (amygdala, hippocampus, thalamus, caudate, putamen, pallidum, nucleus accumbens, and ventral) [37] across both hemispheres. We introduced these 16 ROIs into DK68 atlas, yielding an atlas with 84 ROIs (DK84). In addition, the widely-used automated anatomical labeling atlas with 90 regions (AAL90) [38] was severed as the second parcellation, and the last parcellation subdivided each of the regions of DK68 atlas into four sub-regions according to a parcellation division algorithm [39,40], producing an atlas with 272 regions (DK272).

We then computed all network indices under these three atlases and made statistical inferences on their differences across three groups. The resulting *p*-values are shown in Table 3. All measures except Mod, CC, and EC detected significant differences (*p* < 0.05) between the two patient groups and the control group in three validated parcellations with increasing number of regions. In addition, GE and NS decreased with AD progression in all parcellation schemes while IPF, BNP, CPL, Mod, and EC increased, as shown in Table 3 and Figure 4. Inconsistently, the CC of patient groups decreased in AAL90 atlas, while it presented increasing trends in remaining parcellations. This conflicting result suggests that CC lacks robustness to the characterization of topological deterioration. In contrast, CPL, GE, and NS showed better robustness and sensitivity to the whole-brain parcellation schemes among graph theoretical measures. These results are consistent with existing graph-theoretic findings in [2,35,40]. Moreover, only two persistent features IPF and BNP can recognize (*p* < 0.05) or almost recognize (*p* < 0.1) all group differences across all tested parcellations. Therefore, we could conclude that persistent features are more robust and sensitive than graph-theoretic measures to the characterization of topological deterioration in AD and MCI.

### 3.2. Exploring Other Connectivity Definitions

In addition to the connectivity definition base on fiber count that used in this study, other physiological mechanisms have been adopted to describe WM connections in DTI studies, including distinct fiber count [41], weighted distinct fiber count [41], fractional anisotropy (FA), mean diffusivity, and principal diffusivities ($\lambda_{1},{\;\lambda }_{2},{\;\mathrm{and}\;\lambda }_{3}$), etc. [42]. Among them, FA has usually been applied to define the connection between regions. We validated this physiological parameter and reconstructed WM brain networks. Then, the statistical results of different network indices between groups were obtained. As shown in Table 4, the two persistent homology-based measures demonstrated stronger statistical capability than traditional graph-theoretic measures again.

Moreover, there are various correlation methods to define interregional connectivity. Among them, Pearson correlation is a simple way to study the connection between two related variables, and is often used in the construction of brain networks, especially in the studies based on persistent homology [22,23]. In this study, we used 1 minus Pearson correlation to define the edge weight, so that both positive and negative correlations can be taken into account and the lower weight (i.e., the higher association) represents the shorter path length between regions directly. Although some studies [22,23] including our prior two works [26,34] have applied this approach to connectivity definition, rare research has compared it with other different definition ways, which may yield different results in brain network analysis. For example, in order to consider the non-linearity of the brain data, Spearman correlation may represent a better approach. Then partial correlation computes the complicated relationship between two ROIs while ignores the influence of other ROIs. We validated the two common correlations and used one minus their correlation coefficients to define brain network connectivity. The statistical results are shown in Table 4, where only our previously proposed IPF obtained significant difference between the two patient groups and the control group in both connectivity definitions. In addition, some studies [43] take the absolute value of Pearson correlation instead of 1 minus Pearson correlation as edge weight. Therefore, we validated our dataset by this method and the resulting group differences are shown in Table 4. We found that the experimental results produced by these two approaches of Pearson correlation are basically indistinguishable, by comparing Table 2 and Table 4. Overall, our previously proposed IPF exhibited greatest robustness and sensitivity to different connectivity definitions when comparing the differences between the two patient groups and the control group, while CPL and NS detected significant differences between AD and MCI using Spearman correlation.

### 3.3. Limitations and Future Work

Despite the promising results obtained by applying our suggested persistent features to detect the abnormal structure in whole brain network organization with DTI, there are three important caveats.

First, none of network measures has detected significant difference between AD and MCI under all tested parcellations and connectivity definitions in our dataset, although two persistent features IPF and BNP almost got it (both *p* < 0.1). The clinical symptoms of patients with AD and MCI are so close that sometimes even the clinician can hardly distinguish them. In the future, we may boost the performance of persistent features to discriminate such subtle difference by applying higher dimensional persistent homologies rather than only choosing zeroth persistent homology in this study, because the higher dimensional homology can characterize the more complexed topological structures such as circular holes.

Then, the absence of a functional MRI evaluation represents a limitation of this study because structural and functional changes in WM are not always simultaneously evident. Therefore, the change in metabolism could not be fully explained by structural WM alteration. It might be caused by functional changes, which are not evaluated in this study. Previously, we have applied persistent features to evaluate the functional changes using functional MRI [26] and FDG-PET [34] in our two independent studies. In future, we will integrate structural and functional networks together to investigate the alteration in identical subjects, making the experimental results more interpretable.

Finally, the absence of a longitudinal DTI evaluation is another limitation of this study. We only investigated cross-sectional DTI in this study. However, the change of the longitudinal persistent feature is more meaningful, especially in tracking AD progression and assessing the effectiveness of drugs. In the future, we will investigate time-related dynamics [44] based on tract lengths and longitudinal DTI.

## 4. Materials and Methods

**Figure 5:**
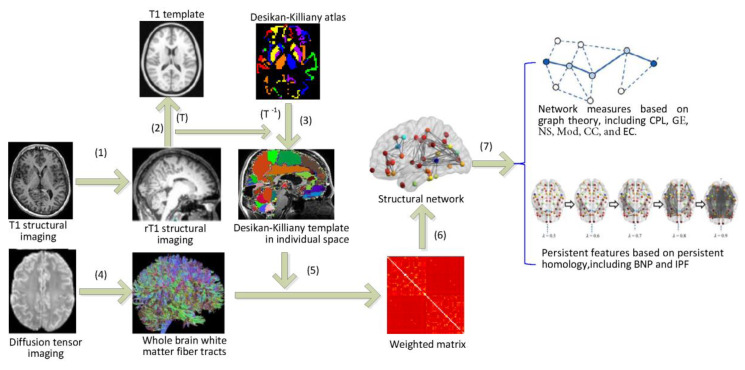
The workflow of brain network analysis based on graph theory and persistent homology. Keys: CPL—characteristic path length; GE—global efficiency; NS—nodal strength; Mod—modularity; CC—clustering coefficient; EC—eigenvector centrality; IPF—integrated persistent feature; and BNP—Betti number plot.

Figure 5 shows the workflow of our study, where the DTI data were preprocessed before constructing WM brain networks, and then some network measures were calculated. The details are described in following subsections.

### 4.1. Subjects and Data Preprocessing

Data used in the preparation of this article were obtained from the ADNI database (adni.loni.usc.edu) [27]. ADNI was launched in 2003 as a public–private partnership led by Principal Investigator Michael W. Weiner, MD. The primary goal of ADNI has been to test whether serial MRI, PET, other biological markers, and clinical and neuropsychological assessment can be combined to measure the progression of MCI and early AD.

DTI can assess the white matter structural connections within the brain, revealing how neural pathways break down in neurodegenerative diseases. We apply our algorithms to 3-Tesla whole-brain diffusion weighted images from the ADNI-2 DTI modality. All participants underwent the whole brain MRI scanning on 3T GE Medical Systems scanners. Standard anatomical T1 weighted SPGR (spoiled gradient recalled echo) sequences were collected in the same session as Diffusion-weighted images (DWI) (256 × 256 matrix; voxel size: 2.7 × 2.7 × 2.7 mm^3^; TR = 9000 ms; scan time = 9 min). There were 46 separate images that were acquired for each diffusion MRI scan: 5 T2-weighted images with no diffusion sensitization (b_0_ images) and 41 diffusion-weighted images (b = 1000 s/mm^2^). DTI preprocessing was performed using PANDA tool (a Pipeline for Analysing braiN Diffusion images) based on FSL5.0 (https://fsl.fmrib.ox.ac.uk/fsl) and the Diffusion Toolkit (Analysis Group, Oxford, UK). The operations of peeling scalp and correction of head movement and eddy current were performed, followed by image resampling. Then all images were registered to standard space, Montreal Neurology Institute (MNI). Finally Gaussian smoothing was applied.

### 4.2. Network Construction

The structural brain network was defined through Desikan-Killiany brain atlas [31] with 68 ROIs. Each ROI was served as a network node. We constructed individual network for each subject using FSL software package following steps (1)–(6) in Figure 5.

(1) Firstly, the T1 image of each subject is registered to its b_0_ image to obtain the rT1 image in each subject space.
(2) Secondly, the rT1 image in the individual space is registered to the T1 template of ICBM-DTI-152 in the MNI space, and the spatial transformation parameter *T* is obtained.
(3) The Desikan–Killiany template in the MNI space is converted into the individual subject space using inverse transform parameter *T*^−1^.
(4) Probabilistic fiber tracking [45] was performed to obtain the white matter fiber tracts in the whole brain tissue of each subject. Each pair of brain regions is assessed using 5000 times of probability tractography and the number of traces that reach both source and target regions is regarded as the connection between them.
(5) We then calculate the weighted matrix *W* (68 × 68) where each element $w_{ij}\;$ measures the similarity of the probability fiber connection patterns between each pair of the brain regions [43]. The edge weight $w_{ij}$ is defined as 1 minus Pearson correlation of fiber connections between them, i.e.,
  (1) $$w_{ij}=1-\frac{cov\left(T_{i},T_{j}\right)}{\sigma_{T_{i}}\sigma_{T_{j}}}$$
  where $\;T_{i},T_{j\;}$ are the fiber connections of the *i*-th and *j*-th brain region to other regions respectively, *cov* is the covariance, σ is the standard deviation, and $\frac{cov\left(T_{i},T_{j}\right)}{\sigma_{T_{i}}\sigma_{T_{j}}}$ is the coefficient of the Pearson correlation.
(6) For each individual, a weighted matrix *W* is treated as a WM brain structural network. The connectivity ranges from 0 to 2 whose value closer to 0 means stronger relationship between a pair of brain regions.

### 4.3. Network Indices

#### 4.3.1. Graph Theoretical Measures

Graph theory is widely used in computer graphics and has been used to describe the topological characteristics of neural networks. The global network attributes based on graph theory characterize the ability of the overall network to transmit information [46]. With the development of brain network science, many network measures have been applied to investigate integration, segregation, centrality, and other characteristics in the structural brain connectome [33,47]. In this paper, the global topological properties of the WM structural brain network are analyzed using CPL [45], GE [48], EC [14], NS [41], Mod [13], and CC [49] in graph theory.

Specifically, CPL and GE are measures of integration. CPL is the average of the shortest paths of any two nodes in a network, and the path length between any pair of nodes is defined as the sum of the lengths of the edges along that path [45]. The calculation formula is:

(2) $$L=\frac{1}{n\left(n-1\right)}\underset{i\neq j\in G}{\sum }L_{i,j}$$

where $L_{i,j}$ is the shortest path length between node *i* and *j*. Conversely, GE is the average inverse shortest path length. Further, NS of a node is defined as the sum of the edge weights connected to the node [41], indicating the strength of a node’s connection with other nodes. Here, we take the average NS over all nodes as the NS of a network. Subsequently, Mod computes the degree to which a network can be subdivided into a set of non-overlapping groups [13]. Then, CC is a measure of segregation and quantifies the extent of interconnected groups in a network. The CC [49] is the average over the whole network of the fraction of a node’s neighbors that are also neighbors of each other. It is defined as:

(3) $$C=\frac{1}{n}\underset{i\in G}{\sum }\frac{\sum_{j,k\in G}{\left(w_{ij}w_{jk}w_{ki}\right)}^{1/3}}{k_{i}\left(k_{i}-1\right)}$$

where $k_{i}$ is the degree of node *i*, and $w_{ij}$ is the edge weight between nodes *i* and *j*. Finally, EC is a measure of the influence of a node in a network, where the higher the assigned score to a node, the more important the node is [14]. In our study, the Brain Connectivity Toolbox (https://sites.google.com/site/bctnet/) was applied to calculate the values of the above network attributes. Before calculating these graph-based indices, each original weighted network was thresholded by only keeping the significant edges (Bonferroni corrected *p* < 0.05) [50], so that the spurious interregional correlations were de-noised.

#### 4.3.2. Persistent Features

**Figure 6:**
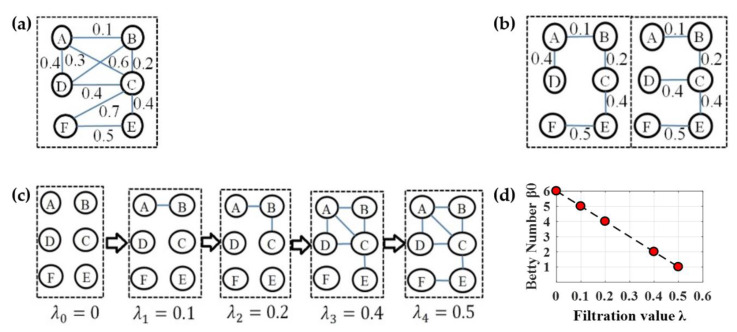
An example illustrating the persistent feature BNP from a weighted network. (**a**) An example network *G*. (**b**) Two possible minimum spanning trees (MSTs). (**c**) Multiscale network by graph filtration at all possible scales. (**d**) The Betti number $\beta_{0}$ plot (BNP).

Persistent homology [51,52] is a method for computing topological features of a space at different spatial resolutions in algebraic topology. More persistent features are detected over a wide range of length and are deemed more likely to represent true features of the underlying space, rather than artifacts of sampling, noise, or particular choice of parameters [53]. The Betti numbers are used to distinguish topological spaces based on the connectivity of *k*-dimensional simplicial complexes [18]. Specifically, the zeroth Betti number $\beta_{0}$, i.e., the zeroth persistent homology refer to the number of connected components, and a connected component in the simplicial complex is a subset of nodes any two of which are connected. From the perspective of the graph decomposition into connected components, we may obtain many possible graph filtrations. In order to determine a maximal graph filtration for quantifying $\beta_{0}\;$ of a general graph, a common approach is single linkage dendrogram (SLD) [22,23,24,25] which is equivalent to constructing a minimum spanning tree (MST) [54]. Thus, we can obtain unique filtrations from the edge weight set of any MST because all possible MSTs of a graph have the identical weight set. As an example, we have two different MSTs in Figure 6b from the graph *G* in Figure 6a, and both have the identical weight set [0.1 0.2 0.4 0.4 0.5], thus the maximal graph filtration at five increasing filtration values 0, 0.1, 0.2, 0.4, and 0.5 is  ℬ(G,0)⊂ℬ(G,0.1)⊂ ℬ(G,0.2)⊂ ℬ(G,0.4)⊂ ℬ(G,0.5) in Figure 6c where $ℬ\left(G,\lambda_{i}\right)$ represents a subnetwork connected by edges with weights less than $\;\lambda_{i}$, and the numbers of connected components $\beta_{0\;}$ are 6, 5, 4, 2, and 1. Finally, the Betti number $\beta_{0}\;$ plot (BNP) over all possible filtration values (Figure 6d) is regarded as a persistent feature of original network in some studies [22,24,26].

Although the BNP shows how the number of connected component varies over different filtration values, it only quantifies the persistent feature in a given state, and the future required persistence information is ignored. Thus, we introduced an aggregation cost to quantify the total required persistence for completing all subsequent evolutions in a nested filtration graph and proposed a novel Integrated Persistent Feature (IPF) in our prior study [26]. The IPF can be understood through the change of total required persistence in the integration process from loose connected components to a fully connected component. In a graph filtration, none of the nodes is connected at the beginning when filtration value λ is zero; the nodes are gradually connected and eventually integrate into one giant component when all nodes are connected. Suppose the total persistence when all components are connected is the target persistence, the total required persistence is getting less with the graph evolution until all components are connected. In this way, the IPF can be thought as the least total required persistence for evolving from current loose connected component to future fully connected component, because the graph filtration is produced by MST in this study. We have previously proven that the IPF plot corresponding to the maximal graph filtration is a monotonically decreasing convergence function.

In clinical settings, doctors prefer single indices as biomarkers because a single neuroimaging index provides a practical reference for evaluating disease progression and for effective treatments. In this study, we define the absolute slope of IPF plot as a network index (IPF index) by the linear regression analysis. The IPF is getting smaller until all connected components are connected and the IPF is equal to zero, thus the proposed IPF index may be thought as the information diffusion rate or the convergence speed of arriving to a fully connected component. Similarly, we define the absolute slope of BNP curve as BNP index. These two persistent features measure the network topology from the perspective of single persistence and total persistence during the weight dynamics. Since all possible thresholding weights are automatically and completely considered in graph filtration, they have better robustness and sensitivity than graph theoretical measures that usually quantify the network topology at a certain thresholding scale. The persistent features are automatically computed under original weighted network, without artificially specifying any threshold schemes.

### 4.4. Statistical Analysis

Statistical analysis of demographic and brain network characteristics were performed using MATLAB R2017a software (Mathworks Inc., Natick, MA, USA). Two nonparametric statistical analyses have been applied in this study. First, the Kruskal–Wallis test [28] was used to evaluate the differences of gender, age, education, and network indices among three groups of AD, MCI and NC. Then, the differences between any two groups of global network indices were determined by 10,000 non-parametric permutation tests [26]. Specifically, for each network index α, assuming that the measurements of the two groups *A* and *B* are $\alpha^{A}=\left\{\alpha_{1}^{A},\alpha_{2}^{A},\cdots ,\alpha_{m}^{A}\right\}$ and ${\;\alpha }^{B}=\left\{\alpha_{1}^{B},\alpha_{2}^{B},\cdots ,\alpha_{n}^{B}\right\}$, we start to calculate the difference of the mean values between the two groups, i.e., $\frac{1}{m}\overset{m}{\underset{i=1}{\sum }}\alpha_{i}^{A}-\frac{1}{n}\overset{n}{\underset{i=1}{\sum }}\alpha_{i}^{B}$. Then we establish the null hypothesis:

(4) $$H_{0}:\frac{1}{m}\overset{m}{\underset{i=1}{\sum }}\alpha_{i}^{A}=\frac{1}{n}\overset{n}{\underset{i=1}{\sum }}\alpha_{i}^{B}$$

and the alternate hypothesis:

(5) $$H_{1}:\frac{1}{m}\overset{m}{\underset{i=1}{\sum }}\alpha_{i}^{A}\neq \frac{1}{n}\overset{n}{\underset{i=1}{\sum }}\alpha_{i}^{B}$$

We further randomly divide $\left\{\alpha_{1}^{A},\alpha_{2}^{A},\cdots ,\alpha_{m}^{A},\alpha_{1}^{B},\alpha_{2}^{B},\cdots ,\alpha_{n}^{B}\right\}$ into two groups of size *m* and *n*, and recalculate the mean differences between the two randomized groups. Repeating this permutation 10,000 times, we finally construct an empirical distribution of the difference based on the above hypothesizes, and the *p* value is determined.

In all statistical analysis of network parameters, a *p* value of less than 0.05 between two groups or a *p-*value of less than 0.01 among three groups is considered to be statistically significant.

## 5. Conclusions

In this study, we focused on investigating the integration and segregation of the WM brain connectome in AD and its precursor MCI using traditional methods of graph theory and our suggesting persistent features. We found significant differences between the patients and controls in many network measures. The patients of AD and MCI presented altered global network properties that were characterized as less integrated and more highly segregated comparing to controls. Moreover, persistent homology-based measures demonstrated stronger statistical capability and robustness than traditionally graph-theoretic methods, suggesting it may be a more sensitive way to detect altered brain structures and to better understand AD symptomology at the network level. This study contributes to an improved mechanistic understanding of disease onset and provides a novel approach to potentially track the progression of AD.

## Figures and Tables

**Table 1:** Demographic information of the subjects.

	AD (*n* = 40)	MCI (*n* = 77)	NC (*n* = 33)	*p*-Value
Male/Female	22/18	42/35	18/15	0.999
Age	72.45 ± 5.60	74.22 ± 6.73	74.18 ± 8.28	0.388
Education	16.1 ± 2.72	15.57 ± 2.67	15.45 ± 2.81	0.443
CDR	≥1	0.5	0	0

(1) Keys: AD—Alzheimer’s disease; MCI—mild cognitive impairment; NC—normal control; and CDR—Clinical Dementia Rating. (2) Data is presented as means ± standard deviations. Statistical group significance was obtained using Kruskal–Wallis test [28].

**Table 2:** Resulting *p*-values of differences across three groups evaluated by different measures on DK68 parcellation.

	IPF	BNP	CPL	GE	NS	Mod	CC	EC
AD vs. MCI	0.084	0.095	0.355	0.235	0.372	0.322	0.308	0.234
AD vs. NC	0.0003	0.001	0.011	0.007	0.011	0.016	0.007	0.049
MCI vs. NC	0.007	0.015	0.014	0.021	0.012	0.024	0.011	0.113
AD vs. MCI vs. NC	0.002	0.003	0.020	0.016	0.020	0.046	0.014	0.470

Keys: AD—Alzheimer’s disease; MCI—mild cognitive impairment; NC—normal controls; IPF—integrated persistent feature; BNP—Betti number plot; CPL—characteristic path length; GE—global efficiency; NS—nodal strength; Mod—modularity; CC—clustering coefficient; and EC—eigenvector centrality.

**Table 3:** Resulting *p*-values of differences across three groups validated by three parcellations.

Parcellation	IPF	BNP	CPL	GE	NS	Mod	CC	EC
Differences on DK84 Atlas								
AD vs. MCI	↑*c*	↑*c*	*ns*	*ns*	*ns*	*ns*	*ns*	*ns*
AD vs. NC	↑*a*	↑*a*	↑*a*	↓*a*	↓*a*	↑*b*	↑*a*	↑*b*
MCI vs. NC	↑*a*	↑*a*	↑*a*	↓a	↓*a*	↑*b*	↑*a*	↑*b*
Differences on AAL90 Atlas								
AD vs. MCI	↑*b*	↑*c*	*ns*	*ns*	↓*c*	↑*c*	*ns*	*ns*
AD vs. NC	↑*a*	↑*a*	↑*b*	↓*b*	↓*a*	↑*b*	↓*b*	↑*b*
MCI vs. NC	↑*a*	↑*a*	↑*b*	↓*b*	↓*b*	↑*b*	↓*c*	↑*b*
Differences on DK272 Atlas								
AD vs. MCI	↑*b*	↑*c*	↑*c*	↓*c*	*ns*	*ns*	*ns*	*ns*
AD vs. NC	↑*a*	↑*a*	↑*b*	↓*b*	↓*a*	↑*b*	↑*c*	↑*b*
MCI vs. NC	↑*b*	↑*b*	↑*b*	↓*b*	↓*b*	↑*c*	↑*b*	↑*c*

(1) Parcellations: DK84, Desikan-Killiany atlas with 84 regions [31]; AAL90, automated anatomical labeling atlas with 90 regions [38]; DK272, subdivision Desikan-Killiany atlas with 272 regions [40]. (2) Keys: AD, Alzheimer’s disease; MCI, mild cognitive impairment; NC, normal controls; IPF, integrated persistent feature; BNP, Betti number plot; CPL, characteristic path length; GE, global efficiency; NS, nodal strength; Mod, modularity; CC, clustering coefficient; EC, eigenvector centrality. (3) Significances: *a*, *p* < 0.01; *b*, 0.01 ≤ *p* < 0.05; *c*, 0.05 ≤ *p* < 0.1; *ns*, *p* ≥ 0.1. (4) Trends: ↑, AD > NC or MCI > NC or AD > MCI; ↓, AD ≤ NC or MCI ≤ NC or AD ≤ MCI.

**Table 4:** Resulting *p*-values of differences validated by different connectivity definitions.

Between-Group	IPF	BNP	CPL	GE	NS	Mod	CC	EC
FA-based connectivity								
AD vs. MCI	0.056	0.073	0.121	0.336	0.231	0.255	0.457	0.436
AD vs. NC	0.009	0.013	0.032	0.016	0.028	0.021	0.047	0.058
MCI vs. NC	0.013	0.022	0.044	0.042	0.025	0.022	0.037	0.086
Spearman Correlation								
AD vs. MCI	0.382	0.246	0.038	0.291	0.036	0.163	0.291	0.046
AD vs. NC	0.048	0.460	0.005	0.225	0.004	0.054	0.307	0.009
MCI vs. NC	0.026	0.302	0.114	0.225	0.111	0.184	0.138	0.147
Partial Correlation								
AD vs. MCI	0.320	0.483	0.406	0.255	0.394	0.433	0.023	0.247
AD vs. NC	0.022	0.045	0.061	0.065	0.063	0.373	0.141	0.064
MCI vs. NC	0.030	0.022	0.068	0.143	0.069	0.421	0.306	0.139
Absolute value of Pearson correlation								
AD vs. MCI	0.072	0.083	0.376	0.287	0.387	0.058	0.321	0.090
AD vs. NC	0.008	0.015	0.012	0.015	0.013	0.020	0.045	0.120
MCI vs. NC	0.012	0.024	0.013	0.043	0.012	0.388	0.071	0.235

Keys: AD—Alzheimer’s disease; MCI—mild cognitive impairment; NC—normal controls; FA—fractional anisotropy; IPF—integrated persistent feature; BNP—Betti number plot; CPL—characteristic path length; GE—global efficiency; NS—nodal strength; Mod—modularity; CC—clustering coefficient; and EC—eigenvector centrality.
